# Singles' similarity preferences in an ideal partner: What, when, and why

**DOI:** 10.3389/fpsyg.2023.1088591

**Published:** 2023-03-02

**Authors:** Jie Liu, Yanyan Zhang

**Affiliations:** ^1^Department of Education, Northeast Normal University, Changchun, Jilin, China; ^2^School of Philosophy and Sociology, Jilin University, Changchun, Jilin, China

**Keywords:** attraction, HEXACO model, ideal partner preference, similarity, personality traits

## Abstract

This study investigated singles' similarity preferences concerning their ideal partner's personality traits, physical attractiveness, and social resources, as well as potential moderators (fear of being single and mate value) and mediators (forecasted satisfaction). With 1,014 Chinese singles, we found that singles preferred their ideal partner to share similarities in the HEXACO traits, physical attractiveness, and social resources, and they preferred higher similarity in Honesty–Humility and Openness to Experience. Fear of being single, mate value, and forecasted satisfaction did not affect similarity preferences concerning Honesty–Humility and Openness to Experience but had some mixed influence over similarity preferences for other features.

## Introduction

In recent years, the topic of ideal partner preference has gained much attention from scholars (e.g., Thomas et al., 2020; Walter et al., 2020; Csajbók and Berkics, 2022). One line of studies primarily examined the similarity preferences for an ideal partner and have generally supported the idea that people prefer their ideal partner to be similar in many attributes, such as personality traits, attitudes, and affects. However, people with intimate relationships tend to adjust their ideal partner preferences based on the characteristics of their current partner (Fletcher et al., 2000; Overall et al., 2006). Liu et al. (2018b) addressed this limitation by recruiting only singles when examining similarity preferences for personality traits in an ideal partner and found that the similarity preference was still held by singles. This study aims to extend the conclusions from Liu et al. (2018b) to show that singles not only have similarity preferences for personality traits but also in physical attractiveness and social resources. In addition, this study explores potential moderators and mediators of such similarity preferences among singles.

### Similarity preferences for ideal partner's features

It is well established that people prefer to have a similar partner from both theoretical and empirical perspectives. From an evolutionary perspective, having a similar partner can promote the passage of one's genes because when two parents share similarities, each parent can contribute more than 50% of their genetic material to their offspring (Thiessen et al., 1997). Niche construction theory indicates that having a similar partner can help people to form congenial and smooth relationships and to construct a desirable environment that fits their needs and facilitates their goals (Laland et al., 2001). From a psychological perspective, pairing with a similar partner is rewarding because similarity can satisfy one's demand for self-affirmation by validating their beliefs and values (Byrne and Clore, 1967) and because similarity can enhance mutual attraction between partners by fostering mutual liking (Condon and Crano, 1988). Besides the theoretical support, some empirical studies have also supported the importance of similarity between partners. For example, partners sharing similar personality traits and/or emotions tend to have more satisfying and stable relationships (Anderson et al., 2003; Luo and Klohnen, 2005; Gonzaga et al., 2007).

Given the importance of having a similar partner, people do depict their ideal partner based on their own characteristics. Past research has shown that people prefer their ideal partner to be similar to them in many aspects, including personality traits, physical attractiveness, attitudes, and values (Botwin et al., 1997; Figueredo et al., 2006; Dijkstra and Barelds, 2008; Watson et al., 2014). However, the relationship status of participants from these studies is either not clear or with some in relationships. Being in a relationship can influence one's ideal preference since people tend to adjust their ideal preference based on their current partner (Fletcher et al., 2000; Overall et al., 2006). Liu et al. (2018b) addressed this issue by only recruiting singles and examining their ideal preference. They found that singles did prefer their ideal partner to share similar personality traits. But Liu et al. (2018b) did not examine whether singles have similarity preferences regarding physical attractiveness and social resources. The current study aims to examine similarity preference among singles not only on personality traits but also on physical attractiveness and social resources. Based on prior literature, we hypothesize that singles prefer their ideal partner to be similar in personality traits, physical attractiveness, and social status (Hypothesis 1).

Previous research not only shows that people prefer their ideal partner to be similar on various attributes but also suggests that similarity preference is particularly pronounced for certain traits. Honesty-Humility and Openness to Experience (from here referred to as Openness) are two potential candidates (Liu et al., 2018b; Liu and Ilmarinen, 2020). For example, Liu et al. (2018b) reported that singles preferred their ideal partner to share a higher similarity in Honesty–Humility and Openness compared to the other HEXACO traits, with participants coming from across China, Denmark, Germany, and the USA. But Liu et al. (2018b) did not examine singles' ideal partner preferences concerning physical attractiveness and social resources. Given that physical attractiveness and social resources are also important when depicting one's future partner as illustrated by ideal standards models, describing the ideal partner from three aspects, including physical attractiveness and social resources (Fletcher et al., 1999; Fletcher and Simpson, 2000), the relative importance of similarity preferences for these two features and personality traits is hard to judge. Some initial observations can be gleaned from studies examining the necessary attributes that people refuse to compromise on when choosing future partners. Li et al. (2002) found that both women and men considered kindness and intelligence as necessities compared to physical attractiveness (which men emphasized more) and social status (which women emphasized more). The two features—kindness and intelligence—nicely mirror some aspects of Honesty–Humility and Openness. Though Li et al. (2002) did not directly examine similarity preferences, their results that kindness and intelligence are prioritized over physical attractiveness and social status are likely to suggest the same when it comes to similarity preferences. Accordingly, we hypothesize that singles have a higher similarity preference concerning Honesty–Humility and Openness compared to the other HEXACO traits, physical attractiveness, and social resources (Hypothesis 2).

### Moderators and mediators of similarity preferences in an ideal partner

Though it is well documented that singles prefer their ideal partner to be similar in many domains, the factors influencing such preferences remain largely unexplored. Liu and Ilmarinen (2020) tackled this issue by exploring the moderation effect of core self-evaluation (i.e., one's overall evaluation of oneself) on singles' similarity preferences in an ideal partner. They found that singles whose overall evaluation of themselves was high preferred their ideal partner to share a higher similarity in Emotionality, Extraversion, Agreeableness, and Conscientiousness, relative to singles whose overall evaluation of themselves was low, suggesting that higher similarity on these traits is deemed as more desirable and only people with more mate-attracting advantages can hope to achieve it. Liu and Ilmarinen (2020) also found that singles' similarity preferences for Honesty–Humility and Openness were not influenced by core self-evaluation, suggesting that similarity preferences for these two traits is less likely to be based on how one evaluates oneself.

In addition to core self-evaluation, other factors are likely to influence singles' similarity preferences in an ideal partner. In this study, we aim to explore not only moderators (i.e., fear of being single and mate value) but also mediators (i.e., forecasted satisfaction) of such preferences.

Fear of being single is defined as “concern, anxiety, or distress regarding the current or prospective experience of being without a romantic partner” (Spielmann et al., 2013, p.1050). Spielmann et al. (2013) showed that people scoring high in fear of being single tend to have lower standards concerning their future partner and are less selective in expressing romantic interest at speed-dating events. Thus, people high in fear of being single might compromise more on their ideal standards.

Mate value describes one's value as a mate to a potential or actual partner (Landolt et al., 1995). Edlund and Sagarin (2010) found that people with high mate value tend to have higher standards when visualizing a future partner (e.g., the partner must be highly attractive, more humorous, livelier, and richer). Accordingly, people high in mate value might be more demanding concerning their ideal standards.

Overall, past research suggests that people low in fear of being single or high in mate value tend to have higher standards concerning their ideal partner. Relating to similarity preferences for personality traits, higher standards indicate higher similarity in Emotionality, Extraversion, Agreeableness, and Conscientiousness (Liu et al., 2018b; Liu and Ilmarinen, 2020). Consequently, we hypothesize that fear of being single and mate value moderate similarity preferences for Emotionality, Extraversion, Agreeableness, and Conscientiousness in the way that people low in fear of being single or high in mate value have higher similarity preference for these traits (Hypothesis 3a). This also applies to physical attractiveness and social resources (Hypothesis 3b). Noticeably, Liu and Ilmarinen (2020) show that similarity preferences for Honesty–Humility and Openness were not affected by moderators. Accordingly, we hypothesize that both moderators had no influence over similarity preferences for Honesty–Humility and Openness (Hypothesis 4).

In addition to examining moderators, we also explore mediators of similarity preferences in an ideal partner. We propose that forecasted satisfaction might be one mediator. Forecasted satisfaction is defined as “anticipated fulfillment and pleasure associated with the relationship in the future” (Lemay, 2016, p.35). Perhaps, people prefer a similar ideal partner due to the belief that they could have good relationships when being with such a partner (Fletcher et al., 2013). Therefore, we hypothesize that forecasted satisfaction mediates singles' similarity preferences for personality traits, physical attractiveness, and social resources (Hypothesis 5).

### The current study

To recap, the current study aims to examine all these hypotheses by recruiting singles who are not currently involved in any kind of intimate relationship. We not only try to replicate previous studies where singles prefer their ideal partner to share similarities concerning personality traits, and such similarity preferences are most pronounced in Honesty–Humility and Openness, but also aim to extend previous studies by examining singles' similarity preferences for physical attractiveness and social resources, determining the relative importance of similarity preferences for these two features and Honesty–Humility and Openness. In addition, we explore two moderators (fear of being single and mate value) and one mediator (forecast relationship satisfaction) of similarity preferences.

## Methods

### Participants and procedure

Singles were recruited from advertisements posted on online social media platforms (e.g., WeChat). Participants were informed that the study would involve participating in an online survey about personality and ideal partner preference. Participants took part in this study voluntarily without monetary compensation but with personalized personality feedback. A total of 1566 participants started our survey and 1078 completed it. Sixty-four participants were deleted because of their patterned response (i.e., reporting 1 or 5 for all personality items). The final sample comprised 1014 participants (81% female), aged between 18 and 46 (*M* = 20.8, *SD* = 2.75).

### Measures

#### Personality

The personality of participants was assessed with the 60-item HEXACO Personality Inventory–Revised (Ashton and Lee, 2009). One sample item is “I would be quite bored by a visit to an art gallery.” These items were answered with a 5-point Likert scale ranging from 1 (*strongly disagree*) to 5 (*strongly agree*). The personality of the ideal partner was measured by an adapted version of the HEXACO inventory about oneself by replacing the first-person pronoun with “my ideal partner” and making grammatical changes only when necessary. Corresponding to the earlier sample item in the measures concerning self-evaluation, the sample item in the ideal partner version is “My ideal partner would be quite bored by a visit to an art gallery.”

#### Physical attractiveness

The physical attractiveness of a participant and their ideal partner was assessed by the vitality–attractiveness dimension from Fletcher et al. (1999). Six descriptions are used, including “nice body” and “attractive.” Participants were instructed to describe their self-perceived physical attractiveness and their ideal partner's physical attractiveness based on these descriptions with a 5-point Likert scale ranging from 1 (*strongly disagree*) to 5 (*strongly agree*).

#### Social status

The social status of participants and their ideal partner was assessed by the status–resources dimension identified by Fletcher et al. (1999). Five descriptions^1^ are used, including “good job” and “financially secure.” Participants were instructed to describe themselves and their ideal partner based on these descriptions with a 5-point Likert scale ranging from 1 (*strongly disagree*) to 5 (*strongly agree*).

#### Fear of being single

Fear of being single was measured by a scale from Spielmann et al. (2013) and was answered on a 9-point Likert scale (1 = *strongly disagree*, 9 = *strongly agree*). One sample item is, “It scares me to think that there might not be anyone out there for me.”

#### Mate value

Participants reported their self-perceived mate value by three items from Landolt et al. (1995). These items include “Men/women notice me” and “Men/women feel attracted to me.” They were measured on a 9-point Likert scale (1 = *strongly disagree*, 9 = *strongly agree*).

#### Forecasted satisfaction

Forecasted satisfaction was measured by an adapted version of the satisfaction scale from Rusbult et al. (1998) and was answered on a 9-point Likert scale (1 = *strongly disagree*, 9 = *strongly agree*). One sample item is “With my ideal partner, our relationship is much better than others' relationships.” An overview of all assessments, datasets, and analyses can be found at https://osf.io/xemyj/.

## Results

### Similarity preference

Table 1 presents the correlations of our main variables. The correlations between self and ideal partner HEXACO traits, physical attractiveness, and social resources ranged from 0.17 to 0.61 (*ps* < 0.001), indicating the existence of similarity preferences. These results remain unchanged after controlling for age and sex (refer to Table 2). Thus, Hypothesis 1, that singles prefer their ideal partner to share similarities in HEXACO traits, physical attractiveness, and social resources, is supported.

**Table 1:** Descriptive statistics and correlations for main variables.

**Variable**	***M***	***SD***	**1**	**2**	**3**	**4**	**5**	**6**	**7**	**8**	**9**	**10**	**11**	**12**	**13**	**14**	**15**	**16**	**17**	**18**	**19**
1. S_HH	3.44	0.64	0.72																		
2. S_EM	3.55	0.62	−0.11^*^	0.71																	
3. S_EX	3.27	0.68	−0.00	−0.11^*^	0.78																
4. S_AG	3.33	0.56	0.25^*^	−0.19^*^	0.25^*^	0.67															
5. S_CO	3.26	0.57	0.14^*^	−0.12^*^	0.22^*^	0.17^*^	0.70														
6. S_OP	3.38	0.67	0.04	−0.12^*^	0.20^*^	0.15^*^	0.15^*^	0.72													
7. S_PA	2.96	0.71	−0.11^*^	−0.17^*^	0.56^*^	0.16^*^	0.19^*^	0.29^*^	0.69												
8. S_SR	3.01	0.73	−0.05	−0.11^*^	0.45^*^	0.16^*^	0.27^*^	0.19^*^	0.54^*^	0.71											
9. P_HH	3.72	0.57	0.61^*^	−0.02	−0.01	0.10^*^	0.08^*^	−0.01	−0.13^*^	−0.05	0.69										
10. P_EM	2.96	0.53	0.02	0.18^*^	−0.02	0.03	−0.07^*^	0.04	0.01	−0.03	−0.07^*^	0.63									
11. P_EX	3.87	0.50	0.07^*^	0.12^*^	0.21^*^	0.09^*^	0.07^*^	0.05	0.12^*^	0.09^*^	0.14^*^	−0.16^*^	0.69								
12. P_AG	3.78	0.49	0.19^*^	0.05	0.12^*^	0.30^*^	0.06^*^	0.05	0.06^*^	0.08^*^	0.31^*^	−0.14^*^	0.37^*^	0.64							
13. P_CO	3.72	0.50	0.03	0.18^*^	0.07^*^	0.02	0.26^*^	0.07^*^	0.04	0.07^*^	0.18^*^	−0.26^*^	0.40^*^	0.35^*^	0.67						
14. P_OP	3.59	0.57	0.08^*^	−0.00	0.12^*^	0.14^*^	0.09^*^	0.56^*^	0.15^*^	0.12^*^	0.16^*^	−0.04	0.33^*^	0.31^*^	0.30^*^	0.72					
15. P_PA	3.93	0.57	−0.13^*^	0.12^*^	0.17^*^	0.05	−0.01	0.09^*^	0.29^*^	0.16^*^	−0.02	−0.12^*^	0.43^*^	0.21^*^	0.27^*^	0.32^*^	0.69				
16. P_SR	4.22	0.65	−0.16^*^	0.22^*^	0.15^*^	0.03	0.08^*^	0.02	0.15^*^	0.17^*^	0.01	−0.21^*^	0.39^*^	0.29^*^	0.46^*^	0.26^*^	0.60^*^	0.85			
17. FoS	4.50	1.61	−0.12^*^	0.31^*^	−0.06	−0.05	−0.10^*^	−0.20^*^	−0.04	−0.02	−0.13^*^	0.14^*^	−0.03	−0.04	−0.08^*^	−0.16^*^	0.02	−0.01	0.69		
18. MA	5.18	1.79	−0.12^*^	−0.10^*^	0.47^*^	0.10^*^	0.21^*^	0.25^*^	0.59^*^	0.40^*^	−0.10^*^	−0.01	0.11^*^	0.04	0.05	0.15^*^	0.20^*^	0.12^*^	−0.03	0.81	
19. FS	7.42	1.27	−0.05	0.06^*^	0.15^*^	0.06	0.09^*^	0.06^*^	0.18^*^	0.15^*^	0.13^*^	0.04	0.26^*^	0.23^*^	0.22^*^	0.17^*^	0.32^*^	0.31^*^	0.11^*^	0.17^*^	0.88

S, Self-evaluation of personality; HH, Honesty–Humility; EM, Emotionality; EX, Extraversion; AG, Agreeableness; CO, Conscientiousness; OP, Openness to Experience; PA, physical attractiveness; SR, social resources; P, ideal partner report personality; FoS, fear of being single; MA, mate value; FS, forecasted satisfaction. Reliabilities are printed in a diagonal line.

* *p* < 0.05.

**Table 2:** Partial similarity preference and higher similarity preference for Honesty–Humility and Openness to Experience.

**Variable**	**Similarity after controlling for gender and age [95% CI]**	**Higher similarity preference for Honesty–Humility [95% CI]**	**Higher similarity preference for Openness to Experience [95% CI]**
Honesty-Humility	0.62 [0.58, 0.65]	–	–
Emotionality	0.29 [0.24, 0.35]	9.36 [0.25, 0.39]	7.54 [0.20, 0.34]
Extraversion	0.21 [0.15, 0.27]	11.34 [0.34, 0.48]	9.88 [0.28, 0.42]
Agreeableness	0.31 [0.25, 0.36]	9.32 [0.24, 0.38]	7.37 [0.19, 0.33]
Conscientiousness	0.26 [0.20, 0.32]	10.19 [0.29, 0.42]	8.46 [0.23, 0.37]
Openness to Experience	0.56 [0.52, 0.60]	–	–
Physical attractiveness	0.31 [0.25, 0.36]	9.06 [0.24, 0.38]	7.59 [0.19, 0.32]
Social resources	0.19 [0.13, 0.25]	11.82 [0.35, 0.49]	10.24 [0.30, 0.44]

Next, we examine Hypothesis 2, that similarity preference for Honesty–Humility and Openness is more important than the other features, by comparing the correlations of these two traits with that of the other features (Liu et al., 2018b). Specifically, we used the method of comparing two non-overlapping correlations from the same group *via* the cocor package in R (Diedenhofen and Musch, 2015). This method is appropriate because all correlations (e.g., the correlation between self-ratings and ideal partner ratings for Honesty–Humility and the respective correlation for Agreeableness) were from the same participants but shared no common variables (e.g., there is no overlap in the items assessing Honesty–Humility and Agreeableness, respectively). Age and sex were also controlled in these comparisons. The results show that the similarity preference is higher for Honesty–Humility and Openness not only relative to the other HEXACO traits but also to physical attractiveness and social resources (7.37 ≤ *z* ≤ 11.82, *ps* < 0.001; refer to Table 2) supporting Hypothesis 2.

### Fear of being single and mate value as moderators

**Figure 1:**
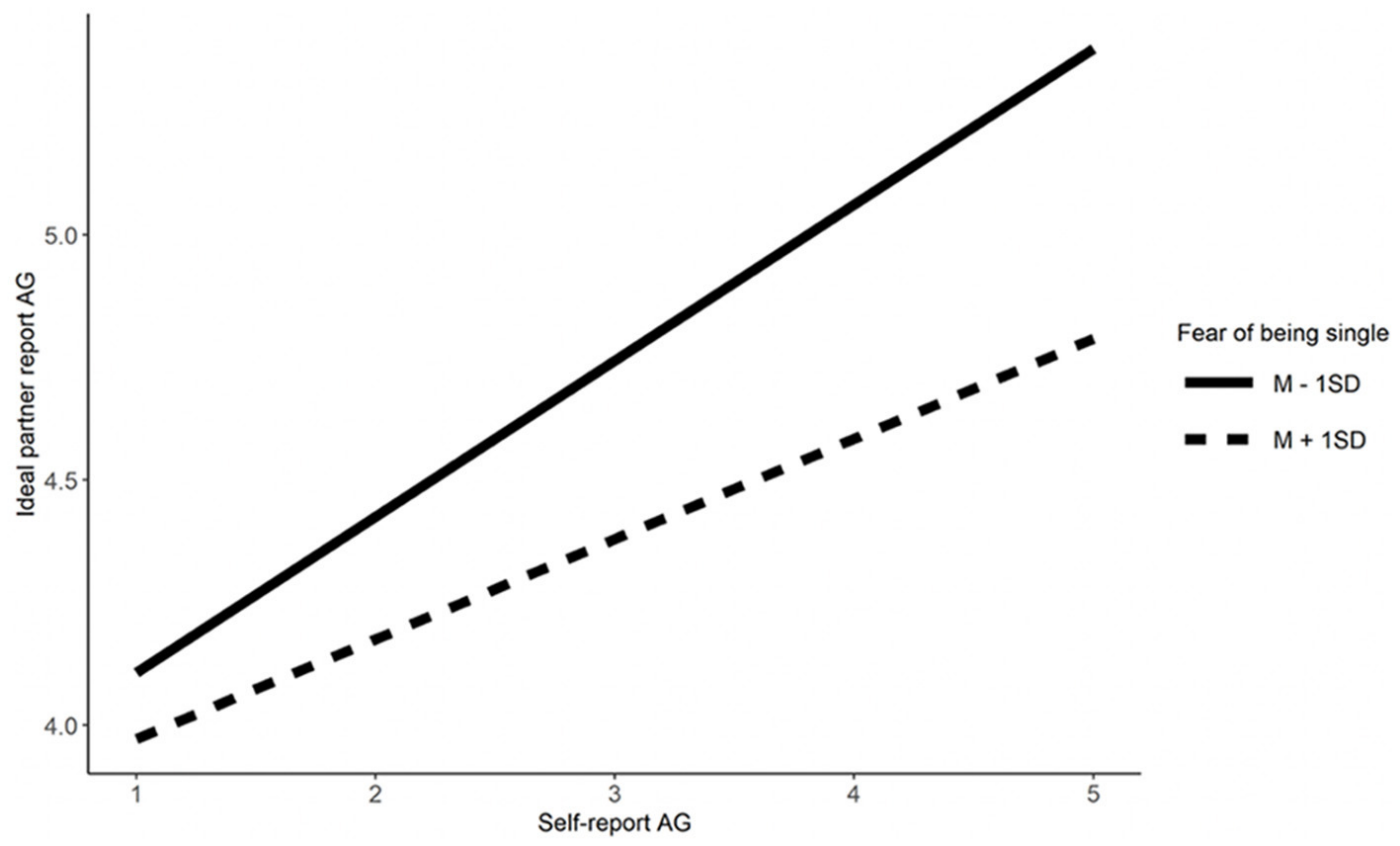
The moderation effect of fear of being single of agreeableness. AG, Agreeableness.

**Figure 2:**
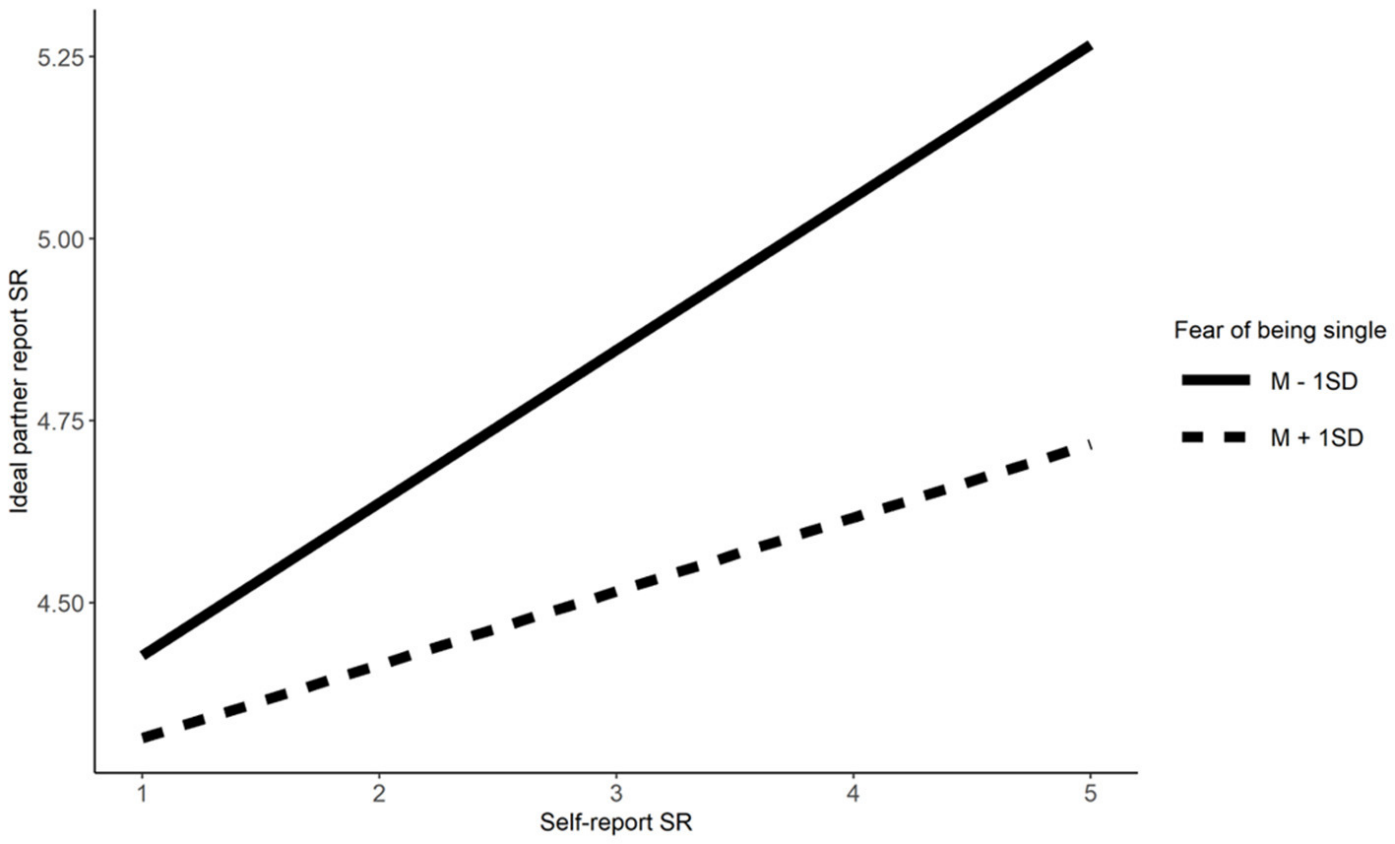
The moderation effect of fear of being single of agreeableness. SR, Social resources.

We examine Hypotheses 3a and 3b, using linear regressions, that fear of being single and mate value moderate similarity preferences for Emotionality, Extraversion, Agreeableness, Conscientiousness, physical attractiveness, and social resources. For each moderator, six regression models were performed with each of the features of self (e.g., self-Emotionality), the moderator (e.g., fear of being single), the interaction (e.g., Emotionality^*^fear of being single), and control variables (i.e., age and gender) as predictors, and the corresponding feature of ideal partner (e.g., ideal partner's Emotionality) as an outcome. The results show that fear of being single moderated similarity preference for Agreeableness and social resources but not on the other characteristics (Refer to Table 3). Simple effects show that singles high in fear of being single showed lower similarity preferences for Agreeableness (*b* = 0.21, β = 0.24, *t* = 5.90, *p* < 0.001) and social resources (*b* = 0.10, β = 0.11, *t* = 2.80, *p* = 0.005) compared to singles low in fear of being single (*b* = 0.32, β = 0.37, *t* = 8.90, *p* < 0.001 for Agreeableness; *b* = 0.23, β = 0.25, *t* = 6.26, *p* < 0.001 for social resources; refer to Figures 1, 2).

**Table 3:** Moderation effects of fear of being single and mate value on HEXACO traits, physical attractiveness, and social resources.

**Predictors**	**Fear of being single**				**Mate value**			
	***b***	β	***t***	***p***	***b***	β	***t***	***p***
**Dependent Variable: P_HH**								
S_HH	0.56	0.63	8.85	< 0.001	0.53	0.59	8.17	< 0.001
Moderator	0.01	0.02	0.14	0.887	−0.01	−0.04	−0.30	0.762
S_HH*Moderator	−0.01	−0.07	−0.48	0.630	0	0.03	0.20	0.844
**Dependent Variable: P_EM**								
S_EM	0.30	0.35	4.42	< 0.001	0.16	0.18	2.37	0.018
Moderator	0.03	0.11	0.66	0.507	−0.06	−0.20	−1.38	0.167
S_EM* Moderator	−0.01	−0.16	−0.80	0.422	0.02	0.21	1.34	0.179
**Dependent Variable: P_EX**								
S_EX	0.13	0.18	2.12	0.035	−0.09	−0.12	−1.54	0.125
Moderator	−0.01	−0.04	−0.26	0.794	−0.14	−0.51	−3.94	< 0.001
S_EX* Moderator	0.01	0.06	0.39	0.694	0.05	0.76	4.34	< 0.001
**Dependent Variable: P_AG**								
S_AG	0.43	0.49	5.71	< 0.001	0.16	0.19	2.23	0.026
Moderator	0.12	0.39	2.28	0.023	−0.06	−0.22	−1.37	0.172
S_AG* Moderator	−0.04	−0.43	−2.31	0.021	0.02	0.28	1.51	0.132
**Dependent Variable: P_CO**								
S_CO	0.11	0.13	1.55	0.121	0.07	0.08	1.01	0.313
Moderator	−0.08	−0.25	−1.60	0.111	−0.09	−0.31	−2.01	0.044
S_CO* Moderator	0.02	0.27	1.60	0.110	0.03	0.40	2.17	0.031
**Dependent Variable: P_OP**								
S_OP	0.44	0.52	7.29	< 0.001	0.36	0.42	5.35	< 0.001
Moderator	−0.04	−0.11	−0.84	0.399	−0.07	−0.21	−1.63	0.104
S_OP* Moderator	0.01	0.08	0.63	0.528	0.02	0.30	1.84	0.067
**Dependent Variable: P_PA**								
S_PA	0.32	0.40	4.83	< 0.001	−0.01	−0.01	−0.13	0.896
Moderator	0.07	0.20	1.65	0.10	−0.11	−0.34	−3.06	0.002
S_PA* Moderator	−0.02	−0.17	−1.22	0.224	0.04	0.62	3.77	< 0.001
**Dependent Variable: P_SR**								
S_SR	0.34	0.38	4.52	< 0.001	−0.13	−0.15	−1.91	0.056
Moderator	0.14	0.35	2.88	0.004	−0.13	−0.34	−3.19	0.001
S_SR* Moderator	−0.04	−0.35	−2.48	0.013	0.05	0.63	4.16	< 0.001

S, Self-evaluation of personality; HH, Honesty–Humility; EM, Emotionality; EX, Extraversion; AG, Agreeableness; CO, Conscientiousness; OP, Openness to Experience; PA, physical attractiveness; SR, social resources; P, ideal partner report personality. Moderator refers to the fear of being single or mate value.

**Figure 3:**
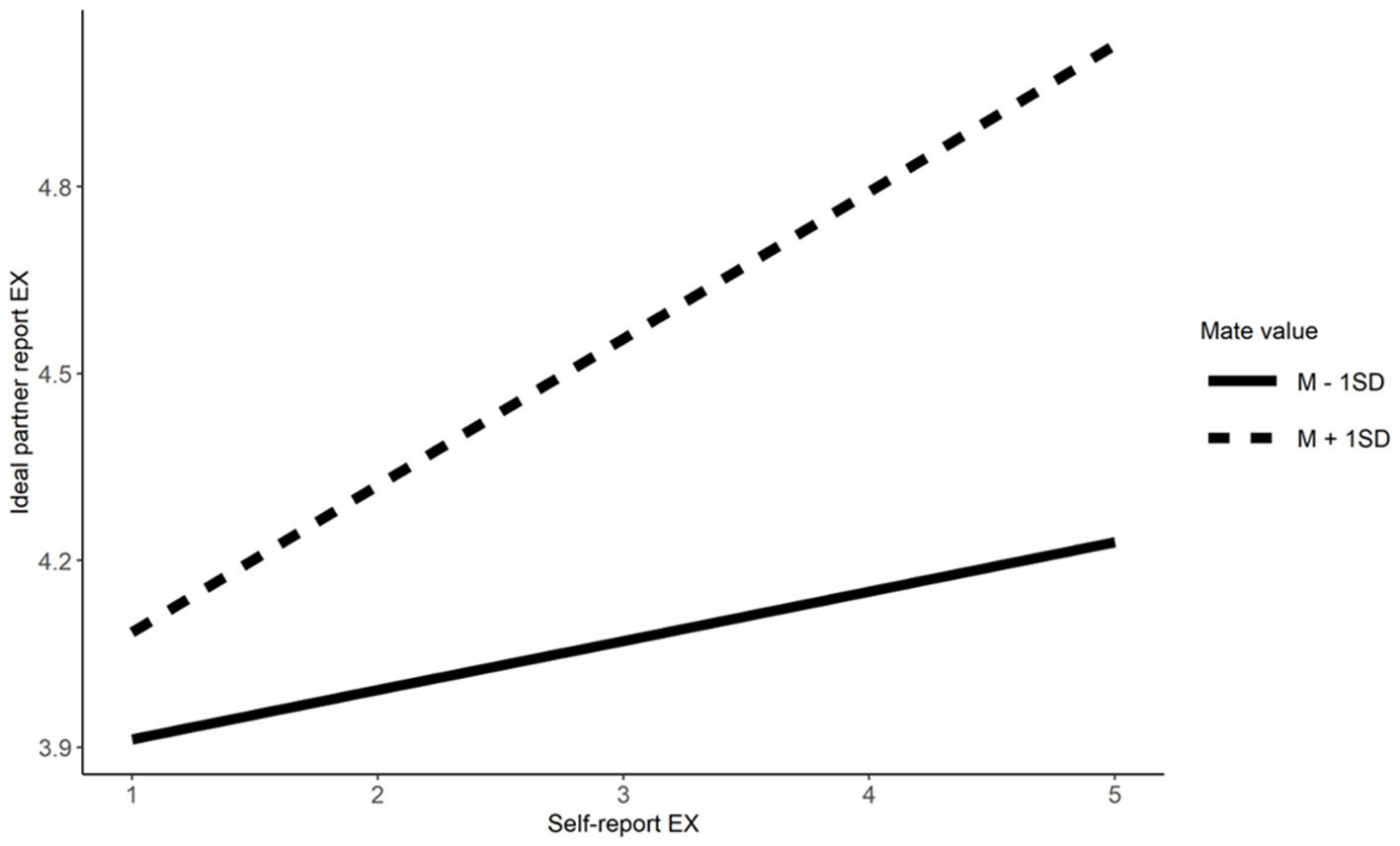
The moderation effect of mate value on similarity preference on extraversion. EX, Extraversion.

**Figure 4:**
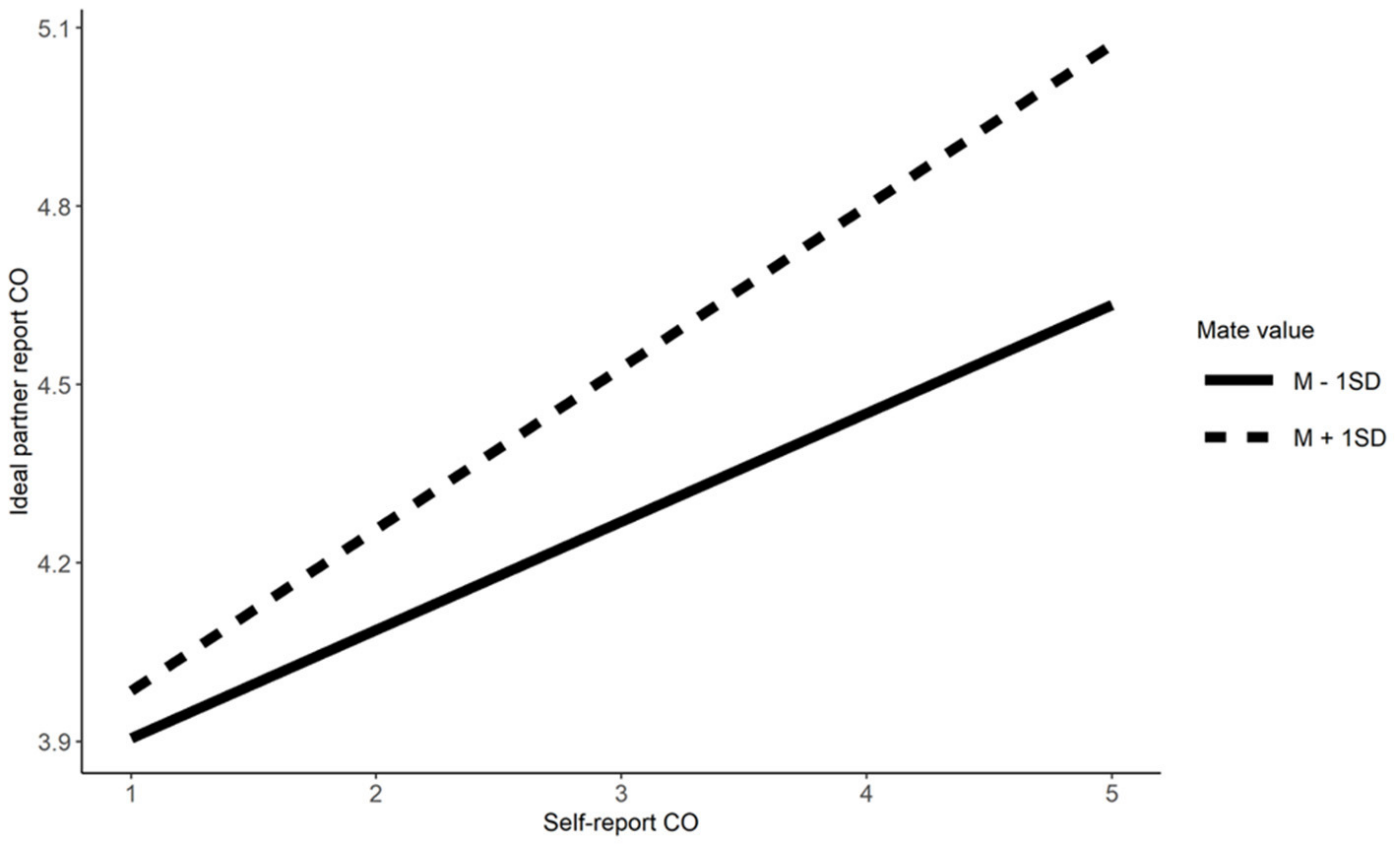
The moderation effect of mate value on similarity preference on conscientiousness. CO, Conscientiousness.

**Figure 5:**
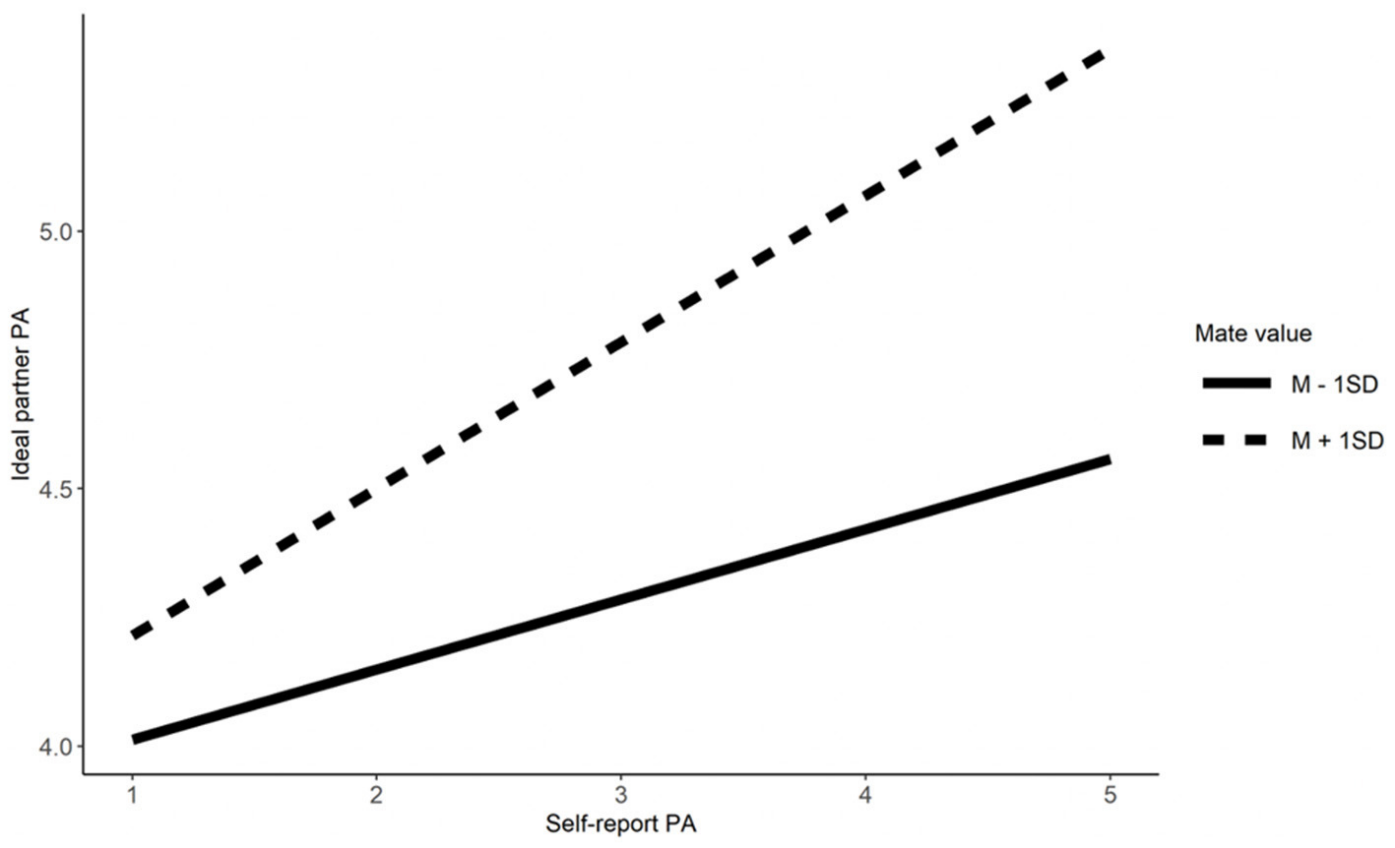
The moderation effect of mate value on similarity preference on physical attractiveness. PA, physical attractiveness.

**Figure 6:**
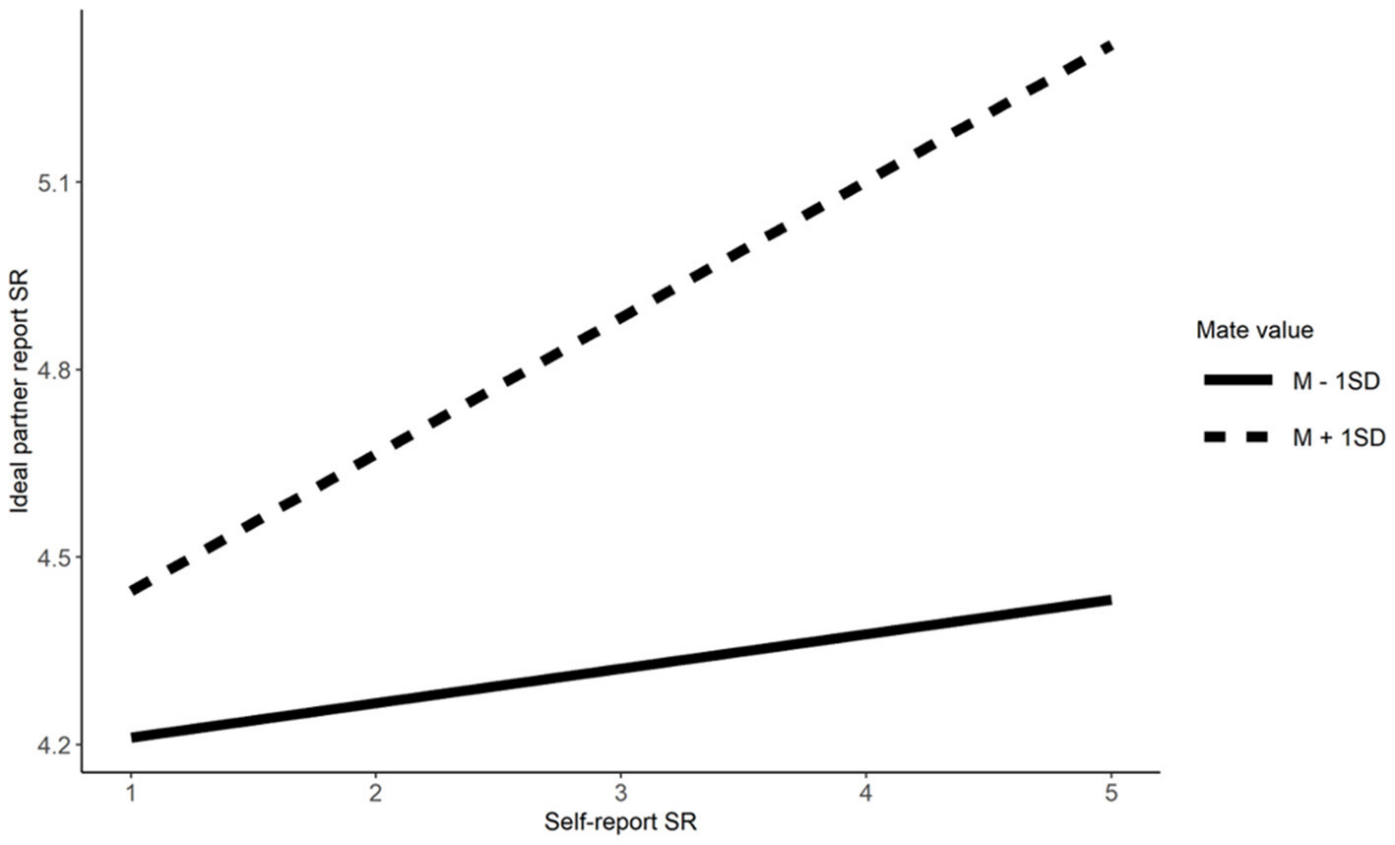
The moderation effect of mate value on similarity preference on social resources. SR, Social resources.

Mate value moderated similarity preferences for Extraversion Conscientiousness, physical attractiveness, and social resources. Simple effects indicate that singles scoring high in mate value have higher similarity preference for Extraversion (*b* = 0.23, β = 0.32, *t* = 7.09, *p* < 0.001), Conscientiousness (*b* = 0.27, β = 0.31, *t* = 7.63, *p* < 0.001), physical attractiveness (*b* = 0.29, β = 0.36, *t*= 8.29, *p* < 0.001), and social resources (*b* = 0.23, β = 0.25, *t* = 6.27, *p* < 0.001), relative to their counterparts low in mate value (*b* = 0.07, β = 0.09, *t* = 2.28, *p* = 0.023 for Extraversion; *b* = 0.17, β = 0.19, *t* = 4.87, *p* < 0.001 for Conscientiousness; *b* = 0.14, β = 0.17, *t* = 3.73, *p* < 0.001 for physical attractiveness, and *b* = 0.04, β = 0.05, *t* = 1.18, *p* = 0.240 for social resources; Refer to Figures 3–6). These results partially supported Hypotheses 3a and 3b.

We then examine Hypothesis 4 that fear of being single and mate value have no impact over similarity preference for Honesty–Humility and Openness. Results from Table 3 show that these moderators did not influence similarity preference for Honesty–Humility and Openness, supporting Hypothesis 4.

### Forecasted satisfaction as a mediator

Hypothesis 5 forecasted that satisfaction may explain the similarity preferences that singles have in their ideal partner, which was examined with mediation models. These mediation models were performed with the Mediation package in R (Tingley et al., 2014). The indirect effect of forecasted satisfaction was significant for similarity preference for Extraversion (β = 0.03, 95% CI [0.01, 0.04]), Conscientiousness (β = 0.01, 95% CI [0, 0.03]), physical attractiveness (β = 0.04, 95% CI [0.02, 0.06]), and social resources (β = 0.04, 95% CI [0.02, 0.06]), indicating that the similarity preferences for these features can be partially explained by forecasted satisfaction, partially supporting Hypothesis 5.

## Discussion

This study examined singles' similarity preferences regarding ideal partner's personality traits, physical attractiveness, and social resources, and we found that singles had similarity preferences for all features, most pronounced in Honesty–Humility and Openness. In addition, we examined the moderation effect of fear of being single and mate value on similarity preference, and the results indicated that neither of these two moderators influenced individuals' preferences for Honesty-Humility and Openness. However, both of the moderators affected similarity preferences for some other features. Specifically, fear of being single moderated similarity preference for Agreeableness and social resources, indicating that singles low in fear of being single preferred their ideal partner to share higher similarity in both features; mate value moderated similarity preference for Extraversion, Conscientiousness, physical attractiveness, and social resources, indicating that singles high in mate value preferred their ideal partner to share higher similarity on these features. Finally, we examined the mediation effect of forecasted satisfaction on similarity preferences and found forecasted satisfaction mediated similarity preference for Extraversion, Conscientiousness, physical attractiveness, and social resources, indicating that expecting a good relationship in the future partially explained why people prefer similarity in these features with an ideal partner.

The results that singles prefer their ideal partner to share similarities in all the HEXACO traits perfectly mirror the conclusion from previous studies (Liu et al., 2018b; Liu and Ilmarinen, 2020). In addition, our results show that similarity preferences are present for physical attractiveness and social resources. Resonating suggestions from Almeida (2004) that people have principles when choosing a romantic partner, our results reflect that similarity between individuals and their ideal partner is one important principle concerning ideal criteria. However, even though it is critical for one to depict a similar partner in a hypothetical way, results from examining established couples have provided a quite mixed picture. Some studies show that couples indeed share similarities with each other (e.g., Watson et al., 2000; McCrae et al., 2008; Leikas et al., 2018), whereas other studies suggest the opposite (e.g., Watson et al., 2014; Liu et al., 2018a, 2022). The seemingly paradoxical phenomenon might be explained by the complication of real-life partner choice. This is because, except for ideal partner preference, there might be some other factors influencing one's actual partner choice, such as the availability of potential partners, family interference, and pursued relationship types. For example, when there are few potential partners available, people are very likely to settle down with partners that do not quite resemble themselves. Future studies could examine how these factors influence similarity preferences in an ideal partner and the relative importance of these factors together with similarity preference when visualizing one's potential future partner.

Furthermore, we found that the similarity preferences were particularly strong for Honesty–Humility and Openness compared to the other four HEXACO traits, which perfectly replicate results from Liu et al. (2018b). Broadly speaking, similarities in Honesty–Humility and Openness can be explained by their close associations with personal values, and people expect to have close relationships with someone who shares their values (Lee et al., 2009). More related to intimate relationships, the emphasis on similarity in Honesty–Humility and Openness might be due to their association with relationship satisfaction and commitment, and people tend to believe that similarity in these two traits is beneficial to relationships (Liu et al., 2022). Furthermore, singles' similarity preferences for Honesty–Humility and Openness outweigh physical attractiveness and social resources, suggesting similarity is more important in key personality traits than more socially desirable features. Future research could use other methods to examine this idea. For example, researchers can use the budget allocation paradigm (e.g., Li et al., 2002) to ask participants to allocate a limited amount of money to indicate similarity preference for HEXACO traits, physical attractiveness, and social resources and observe what feature people allocate the largest portion of the money. Furthermore, it is unclear how individuals make a trade-off between competing preferences such as preference for similarity in certain traits and preference for an absolute level of various characteristics, such as physical attractiveness. Actually, similarity in personality in established heterosexual couples tends to be quite low, even in Honesty–Humility and Openness (Liu et al., 2018a, 2022). Accordingly, people may trade the similarity of these two traits with other individual features when choosing a real-life partner. It would be interesting to examine whether men tend to trade similarities in Honesty–Humility and Openness with physical attractiveness while women trade similarities in these two traits with social resources, as men and women are shown to emphasize different aspects in their future partner from evolutionary perspectives (Buss, 1989). Future studies could further explore these issues.

Though Liu et al. (2022) found that similarity in Honesty–Humility and Openness in intimate couples from China tends to be quite low, a recent study by Kandler et al. (2019) has shown the opposite. Indeed, Kandler et al. (2019) found that their participants, 228 German couples, presented quite a high similarity in Honesty–Humility (*r* =0.225) and Openness (*r* =0.277). Therefore, similarities in Honesty–Humility and Openness might be different depending on different relationship types (e.g., married vs. unmarried) and different cultures (e.g., collectivism vs. individualism). For example, it is possible that similarities in Honesty–Humility and Openness in married couples is more significant than in unmarried intimate couples. Future studies could further explore these possibilities.

We found that similarity preferences for Honesty–Humility and Openness was not moderated by fear of being single, and mate value also indirectly reflects the particular importance of similarity in these two traits. These results nicely echo the conclusion from Liu and Ilmarinen (2020) that similarity preferences for these two traits was not moderated by core self-evaluation. Together, these results indicate that singles' similarity preferences for Honesty–Humility and Openness are quite strong and immune from potential moderators relating to individual differences. Future research could examine whether social factors, such as the availability of potential partners and relational factors, such as relationship types (e.g., long-term vs. short-term relationships), have an influence on similarity preferences for these two traits.

The moderation hypotheses are only partially supported. Fear of being single and mate value had mixed moderation effects on Extraversion, Agreeableness, Conscientiousness, physical attractiveness, and social resources, but overall they suggest that people low in fear of being single or high in mate value are more demanding concerning similarity preferences in an ideal partner. These results not only echoed the results from Liu and Ilmarinen (2020) that people with high self-evaluation tend to have high ideal standards but also confirmed that some personality traits (e.g., Extraversion, Agreeableness, and Conscientiousness) are more socially desirable.

The mediation effects of forecasted satisfaction were only supported by similarity preference for Extraversion, Conscientiousness, physical attractiveness, and social resources, indicating that expecting a satisfying relationship in the future is the reason why singles prefer a similar partner. This actually mirrors the main idea of niche construction theory that people are motivated to build an environment that is congenial, fluent, and low in conflict (Laland et al., 2001). In the setting of an intimate relationship, our study shows that the reason why people initially prefer to have a similar partner is because they presume that such a partner can help to form a satisfying relationship in the future. For example, if Sally is high in Extraversion, she would like to have a partner who is high in Extraversion; this is because she could easily imagine a happy relationship with such a partner, not only more pleasures and joys (e.g., going to parties together) but also fewer disagreements and conflicts (e.g., negotiating being alone vs. socially active) in future. However, since the partial mediation models suggest the existence of other mediators, future research could explore other potential mediators, such as intimacy, responsiveness, and commitment.

The current study also has some limitations. First, most participants in our study were female, which may prevent us from generalizing our conclusions to more gender-balanced samples. Future research should strive for a gender-balanced sample to further examine this topic. Second, our participants are relatively young, meaning our study is limited in its representation of older individuals. Future research could explore whether older singles still exhibit the same patterns. Third, we mainly used the method where a participant only reports information about themselves to collect our data. Accordingly, our results might be affected by some response biases, such as acquiescence response style, social desirability bias, and self-enhancement bias. For example, self-rated physical attractiveness might not be that objective due to self-enhancement bias, and people are likely to think of themselves as more attractive than they actually are (Epley and Whitchurch, 2008). Future researchers could combine self-rated and other-rated methods to measure these variables in a more comprehensive and objective way to further test these hypotheses. Finally, though the moderation and mediation effects in our study add some important insights to the current literature concerning similarity preference in an ideal partner, they are only partially supported. Future research could continue examining these moderation and mediation effects to further test their robustness.

## Conclusion

Overall, this study examined singles' similarity preferences concerning their ideal partner's personality traits, physical attractiveness, and social resources, as well as potential moderators (fear of being single and mate value) and mediators (forecasted satisfaction). Our results show that singles had similarity preferences in their ideal partner for the HEXACO traits, physical attractiveness, and social resources. This preference was higher for Honesty–Humility and Openness to Experience relative to the other features. In addition, fear of being single, mate value, and forecasted satisfaction did not affect similarity preference for Honesty–Humility and Openness to Experience but had some mixed influence over similarity preferences for other features.

## Data availability statement

The raw data supporting the conclusions of this article will be made available by the authors, without undue reservation.

## Ethics statement

The studies involving human participants were reviewed and approved by IRB Board, Department of Psychology, School of Philosophy and Sociology, Jilin University. The patients/participants provided their written informed consent to participate in this study.

## Author contributions

JL prepared the manuscript and performed the analyses. JL and YZ helped in interpreting the results and editing the manuscript. All authors contributed to the article and approved the submitted version.

## Footnotes

^1^The original status–resources dimension has six descriptions, with “appropriate ethnicity” included. We excluded this one because it is not meaningful in China.
